# On the move

**DOI:** 10.7554/eLife.34901

**Published:** 2018-02-21

**Authors:** Sandra L Martin

**Affiliations:** Department of Cell and Developmental BiologyUniversity of Colorado School of MedicineAuroraUnited States

**Keywords:** LINE-1, retrotransposon, cell cycle, ORF1p, ORF2p, Human, Virus

## Abstract

The mechanisms by which a retrotransposon called LINE-1 duplicates itself and spreads through the human genome are becoming clearer.

**Related research article** Mita P, Wudzinska A, Sun X, Andrade J, Nayak S, Kahler DJ, Badri S, LaCava J, Ueberheide B, Yun CY, Fenyö D, Boeke JD. 2018. LINE-1 protein localization and functional dynamics during the cell cycle. *eLife* **7**:e30058. doi: 10.7554/eLife.e30058

**Related research article** Taylor MS, Altukhov I, Molloy KR, Mita P, Jiang H, Adney EM, Wudzinska A, Badri S, Ischenko D, Eng G, Burns KH, Fenyö D, Chait BT, Alexeev D, Rout MP, Boeke JD, LaCava J. 2018. Dissection of affinity captured LINE-1 macromolecular complexes. *eLife* **7**:e30094. doi: 10.7554/eLife.30094

Over half of our DNA comes from retrotransposons, genetic elements that independently multiply and spread through the genome (de Koning et al., 2011). In mammals, a retrotransposon called LINE-1 is responsible for most of these multiplication events, either duplicating itself or lending its 'photocopying' machinery to other genetic elements. These phenomena are made possible by a mechanism called reverse transcription: the DNA sequence of the retrotransposon is transcribed into RNA, which is then reverse transcribed back into DNA and inserted into the genome. While most of the new LINE-1 elements are no longer capable of retrotransposition, there is a small number of active LINE-1 elements that cause variation and disease among humans, and even among cells within a given person. Analysis of LINE-1 sequences in various mammalian species reveals a long-running evolutionary arms race between selective pressures that maintain retrotransposition in the element, and host proteins that try to repress retrotransposition by interacting with LINE-1 at various points in its life cycle (see Goodier, 2016 for a comprehensive review).

The life cycle of LINE-1 begins with the transcription of its DNA sequence in the nucleus. The resulting mRNA is then exported to the cytoplasm, where it is translated to produce two proteins: ORF1p, which forms trimers and binds mRNA, and ORF2p. The latter, which is translated less efficiently than ORF1p, acts both as an endonuclease (which can cleave bonds in DNA) and as a reverse transcriptase. ORF1p, ORF2p and LINE-1 mRNA then assemble to form a complex that must gain access to the nucleus to reach the DNA. Once it does, the ORF2p protein cleaves one of the strands, and the mRNA molecule undergoes reverse transcription to make a length of complementary DNA contiguous with the host DNA. Despite the apparent simplicity of this mechanism, numerous questions about LINE-1 remain: how does the ORF1p/ORF2p/LINE-1 mRNA complex, which is made in the cytoplasm, gain access to genomic DNA, which is in the nucleus? Is it actively transported, or does it gain access by waiting for the membrane around the nucleus to break down naturally during cell division? And at what steps in the LINE-1 replication cycle do cellular proteins interact to help or block retrotransposition?

Now, in two papers in eLife, Paolo Mita and Jef Boeke of NYU Langone Medical Center, John LaCava of Rockefeller University and co-workers in the United States and Russia report how they have used a combination of state-of-the-art affinity capture, mass spectrometry and advanced imaging techniques to examine how and when LINE-1 replication intermediates get access to the nucleus, and to identify some of the cellular proteins that interact with ORF2p and have an impact on the retrotransposition process.

In the first paper, LaCava and co-workers – including Martin Taylor of Massachusetts General Hospital and Ilya Altukhov of the Moscow Institute of Physics and Technology as joint first authors – report the existence of two groups of proteins that interact with ORF2p (Taylor et al., 2018). The first set includes ORF1p and several RNA binding proteins that are typically found in the cytoplasm: although these proteins attach to the same RNA as ORF2p, they do not bind ORF2p directly. The second set is formed of nuclear proteins, including some that are involved in DNA synthesis and repair: it is likely that these proteins form a complex directly with ORF2p. Surprisingly, ORF2p had not been seen in the nucleus before. However, in previous experiments dividing cells were lost: by growing cells under conditions suitable to detect mitotic cells, Taylor et al. are able to see ORF2p in the nucleus.

Significantly, ORF2p is only found in the nucleus if ORF1p is expressed from the same RNA, suggesting that the latter somehow shepherds the complex into the nucleus. If the reverse transcriptase activity of ORF2p is inactivated by mutation, its association with ORF1p and various RNA binding proteins is enhanced. This suggests that reverse transcription displaces both ORF1p (see also Naufer et al., 2016) and the other RNA binding proteins before the complex that contains ORF2p and the nuclear proteins can form.

In the second paper Mita and co-workers report when these events occur in the cell cycle (Mita et al., 2018). While ORF1p is readily detected in the cytoplasm of nearly all cells, it is present with ORF2p and LINE-1 RNA in just a small subset of these. ORF1p is found in the nucleus in an even smaller number of cells going through the G_1_ phase of the cell cycle (which is when the cell grows) but not in any of the other stages. In contrast ORF2p is found in nuclei with and without ORF1p, and persists longer into other phases of the cell cycle. These results point to the retrotransposition complex getting access to the genome during mitosis, when the nuclear membrane disappears.

Mita et al. go on to use advanced imaging techniques to study how often new LINE-1 elements are created and inserted into the genome at different stages of the cell cycle. A functional LINE-1 is marked by a fluorescent reporter gene that can only be detected after retrotransposition is completed. A technique called flow cytometry quantifies how often this gene is activated. The results show that most of the insertion events take place during the S phase, when the cell replicates its DNA. This is true both for cells that divide freely and for those that are chemically forced to replicate at the same time.

When combined with previous results, the two new papers paint a complicated picture that involves a range of complexes: some of these are intermediates that aid the process of retrotransposition, but others prevent it. Only a small fraction of ORF1p/LINE-1 RNA complexes are able to squeeze through the bottleneck that pushes most of the ORF1p into a dead-end cytoplasmic complex, and to ultimately acquire ORF2p. The resulting complex – which now contains all three of the LINE-1 components needed for retrotransposition – must survive in the cytoplasm until the nuclear envelope breaks down during cell division, thus providing access to the cell's DNA. The complex again must persist in the nucleus until the S phase, when replication proteins and new nucleotides become available. Reverse transcription displaces ORF1p, allowing ORF2p to form a new complex with DNA replication and repair factors – some of which may facilitate but others interfere with successful completion of LINE-1 replication. Although many details remain to be learned, the work described in the papers by Mita et al. and Taylor et al. represents a giant step towards understanding LINE-1 retrotransposition.
